# Intelligent Fault Diagnosis of Planetary Gearbox Across Conditions Based on Subdomain Distribution Adversarial Adaptation

**DOI:** 10.3390/s24217017

**Published:** 2024-10-31

**Authors:** Songjun Han, Zhipeng Feng, Ying Zhang, Minggang Du, Yang Yang

**Affiliations:** 1 School of Mechanical Engineering, University of Science and Technology Beijing, Beijing 100083, China; songjun0038@163.com (S.H.); fengzp@ustb.edu.cn (Z.F.); 2 China North Vehicle Research Institute, Beijing 100072, China; mgdu@noveri.com.cn (M.D.); yangyang@noveri.com.cn (Y.Y.)

**Keywords:** planetary gearbox, intelligent fault diagnosis, vibration signal, adversarial training mechanism

## Abstract

Sensory data are the basis for the intelligent health state awareness of planetary gearboxes, which are the critical components of electromechanical systems. Despite the advantages of intelligent diagnostic techniques for detecting intricate fault patterns and improving diagnostic speed, challenges still persist, which include the limited availability of fault data, the lack of labeling information and the discrepancies in features across different signals. Targeting this issue, a subdomain distribution adversarial adaptation diagnosis method (SDAA) is proposed for faults diagnosis of planetary gearboxes across different conditions. Firstly, nonstationary vibration signals are converted into a two-dimensional time–frequency representation to extract intrinsic information and avoid frequency overlapping. Secondly, an adversarial training mechanism is designed to evaluate subclass feature distribution differences between the source and target domain. A conditional distribution adaptation is employed to account for correlations among data from different subclasses. Finally, the proposed method is validated through experiments on planetary gearboxes, and the results demonstrate that SDAA can effectively diagnose faults under crossing conditions with an accuracy of 96.7% in diagnosing gear faults and 95.2% in diagnosing planet bearing faults. It outperforms other methods in both accuracy and model robustness. This confirms that this approach can refine domain-invariant information for transfer learning with less information loss from the sub-class level of fault data instead of the overall class level.

## 1. Introduction

Planetary gearboxes are vital components of electromechanical systems, e.g., vehicles and wind turbines. A malfunction of the gearbox can result in system breakdowns and potentially serious safety incidents. Thus, accurately diagnosing faults in planetary gearboxes is crucial for maintaining the safe and reliable operation of this equipment [1,2,3].

In decades, researchers have extensively studied the fault diagnosis of planetary gearboxes. For example, Zhao et al. [4] developed a gear mesh stiffness model that integrates friction and fractal contact theory, aiming to analyze the relationship between friction factors and fractal parameters. This model provides insights into how these factors interact and influence gear performance. Xiang et al. [5] introduced a lateral torsion dynamics model tailored for multi-stage gear systems. This model reveals the mechanisms behind spalling and the progression of faults within these systems. Their comprehensive study also explores the mapping relationship between various gear fault modes and their dynamic characteristics, providing a significant reference point for future fault diagnosis research. This exploration helps in understanding how different fault modes impact the dynamic behavior of gear systems, thereby aiding in the development of diagnostic techniques. Feng et al. [6,7] developed AM-FM analysis models for vibration signals at gear meshing points. They derive the characteristic frequency formula for gear faults on the basis of Fourier transform and identify the spectral characteristics of typical faults. Jiang et al. [8] proposed a novel decomposition scheme for variational mode decomposition. This method resolved the problem of uncertain decomposition modes and balance parameter selection. Traditional fault diagnosis methods require the assistance of expertise knowledge for feature extraction, which limits their applications greatly. To overcome these problems, Zhao et al. [9] introduced a novel fault diagnosis method for rolling bearing. This method focuses on reducing the dimensionality of the features. It can capture more intrinsic fault information, which allows for a more precise identification of faults by isolating the most relevant information from potentially noisy or irrelevant data. Yu et al. [10] developed an innovative data-driven multigranulation model for fault diagnosis of planetary gearboxes. By leveraging multiple granular perspectives, this model allows for a detailed and nuanced analysis. To minimize dependence on labeled data, Wu et al. [11] proposed a hybrid classification model based on an autoencoder. The model can be trained unsupervised with unlabeled data. Zhang et al. [12] presented an innovative adaptive loss-weighted meta-residual network specifically designed to address the challenge of label noise interference in fault diagnosis. This advanced network dynamically adjusts the weights assigned to faulty data during the training process, allowing the model to better distinguish between accurate and noisy labels. Yin et al. [13] embedded cosine loss optimization into an LSTM network for gear fault diagnosis in wind turbines, enhancing the diagnostic accuracy over traditional LSTM networks. Deep learning-based diagnostic methods construct end-to-end models from raw data to equipment health status, automatically extracting useful features from complex data to accurately identify equipment health. These methods reduce dependence on expert knowledge and provide robust technical support for intelligent fault diagnosis.

Traditional data-driven methods for intelligent fault diagnosis often operate under the assumption that training and test data (source-domain and target-domain data) are independently and identically distributed. However, in real application, the operation of a planetary gearbox exhibits time-varying characteristics under varying speeds or loads. These fluctuations result in an uneven distribution of monitoring data, potentially indicating different health states even when the actual health condition remains the same. This cause discrepancies between source-domain and target-domain data, thus requiring diagnostic models to be highly adaptable and capable of accurately interpreting and analyzing signals that may change in unpredictable ways, while overlooking the discrepancies could undermine the performance of the proposed method. This complexity underscores the need for transfer learning techniques.

Transfer learning facilitates the application of knowledge gained from one domain (source-domain) to improve diagnostic performance in a different, but related, domain (target-domain) [14]. The source domain consists of labeled training data $D_{S}=\left\{x_{i}^{S},y_{i}^{S}\right\}$, which provides necessary information for initial model training. In contrast, the target domain contains unlabeled monitoring data $D_{T}=\left\{x_{j}^{T}\right\}$. The model must analyze and interpret without predefined labels. To bridge the gap between domains, domain adaptation has been proposed as a crucial technique within transfer learning. Li et al. [15] and Zhu et al. [16] proposed a range of metric learning algorithms to evaluate and quantify the differences of features between source and target domains. These approaches focused on minimizing the distance between variations within the same class (intra-class variation) while maximizing the distance between variations of different classes (inter-class variation). This strategy significantly enhanced the robustness and reliability of diagnostic models by ensuring that the features extracted were more distinct and discriminative. Deng et al. [17] incorporated a range of attention mechanisms into adversarial networks, allowing the networks to focus on the most relevant features across different domains. This integration ensures that the learned features were applicable regardless of domain discrepancies. To make an alignment of data distribution across domains, joint distribution adaptation (JDA) [18,19,20] was proposed to evaluate the joint distribution discrepancy between source and target tasks for cross-domain monitoring data in fault diagnosis. Current methods enable the model to adapt the knowledge gained from the labeled source domain to the unlabeled target domain, thus can more effectively generalize across different operational conditions and datasets, leading to more robust and accurate fault detection.

**Figure 1:**
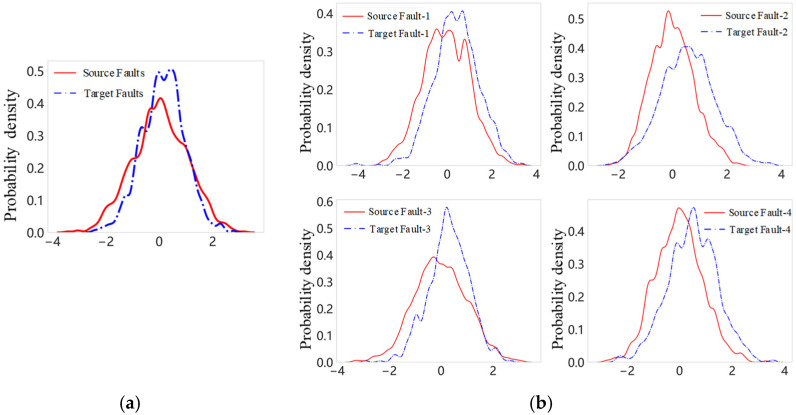
Diagram of data distribution adaptation among different fault classes: (**a**) averaged distribution of faults; (**b**) data distribution of different faults.

With unlabeled data in the target domain, E. Tzeng et al. [21] proposed an adaptation layer and an additional domain confusion loss for convolutional neural networks to learn a representation that is both semantically meaningful and domain invariant. Ganin et al. [22] proposed a domain adaptation method by augmenting a network with few standard layers and a simple new gradient reversal layer. The approach can effectively learn deep features that are invariant with respect to the feature shift between the domains. Yu et al. [23] proposed a dynamic adversarial adaptation network for transfer learning. These methods effectively learn features with unlabeled data. However, they only align overall distributions of domain data by adversarial training or metrics like maximum mean discrepancy (MMD), and fail to capture significant variabilities of individual class of fault data. By only focusing on the overall distribution, as illustrated in Figure 1a, traditional domain adaptation methods run the risk of neglecting fault-specific information that is critical for accurate diagnosis. Certain features that are highly relevant to identifying a specific type of fault might be averaged out when aligning the global data distributions. This lack of focus on subclass-level variations can reduce the model’s ability to precisely distinguish between different fault types and lead to suboptimal performance in fault diagnosis tasks.

In real-world applications, different types of planetary gearbox faults can exhibit distinct characteristics, leading to variations in their data distributions. As shown in Figure 1b, the subclass fault data of a planetary gearbox does not follow a single, unified distribution but instead adheres to different data distributions for each fault type. While traditional domain adaptation approaches can transfer general knowledge from related domains, they do not effectively account for the nuances of subdomain-specific data distributions. This limitation highlights the need for methods that not only align global distributions but also consider the intra-domain variations and the conditional distributions of different fault types, ensuring that the model retains the fault-specific information necessary for accurate and reliable diagnosis. To tackle the aforementioned challenges, this paper introduces a subdomain distribution adversarial adaptation approach (SDAA) for identifying faults of planetary gearboxes under cross conditions. This method evaluates the subclass data discrepancy between the source domain and target domain. By assessing feature differences between various subclass faults, SDAA seeks to align the feature distribution spaces between source and target tasks more closely. It individually aligns each type of fault data before making joint adjustments. The shared structures or patterns are identified for transfer from the source domain to the target domain. This method ensures that the subclass-level alignment is more accurate by considering the relationships between features within each fault subclass. From a network structure perspective, the SDAA method employs deep residual networks to construct a dual-stream feature extraction architecture. This architecture simultaneously learns feature information from both source and target tasks. Local difference adversarial evaluation is used to measure feature differences among relevant subclass faults in different tasks, thereby mining subclass fault feature information and reducing model bias. Furthermore, the method uncovers the correlation of transferrable information by assessing conditional distribution differences between source and target subclass fault data. It identifies and leverages the relationships and similarities between different types of fault data. This detailed analysis allows the method to transfer relevant information more accurately from the source domain to the target domain. Through this process, the method ensures that the diagnostic model is well-equipped to handle variations and discrepancies in fault data across different operational conditions with the enhanced robustness and accuracy.

In real application, the signals of planetary gearbox tend to exhibit time-varying characteristics. Using the time-domain signal as the input of SDAA for fault diagnosis could overlook the intrinsic and dynamic characteristics of signal. To solve this problem, the proposed method uses time–frequency analysis as the input of deep learning. The short-time Fourier transform (STFT) is used to extract time–frequency features from vibration signals. It can characterize the non-stationary condition effectively. STFT captures the time-varying nature of frequency components and amplitudes, thereby mitigating the challenges of frequency overlapping and enhance the distinguishability of features. The time–frequency feature can provide more intrinsic information on health state. Thus, the SDAA adapts the framework of network with the time–frequency representation as the input.

The main contributions of this paper are summarized as follows.

(1) A novel framework incorporating the time–frequency representation construction and subdomain distribution adversarial adaptation diagnosis method is proposed for the fault diagnosis of planetary gearboxes. It allows the model to generalize better across a wide range of operating conditions by aligning the subclass and overall class of data simultaneously, which enhances the fault diagnosis performance.
(2) Time–frequency representation is adopted as the input of deep learning to provide intrinsic information of health state. The STFT is applied to extract time–frequency representation from vibration signals. It can reflect the variation of frequency components and amplitudes over time, which can characterize the non-stationary condition of planetary gearbox more confidently.
(3) Local difference adversarial evaluation is used to discover the correlation of faults information by assessing conditional distribution differences between source and target subclass fault data. It can handle variations and discrepancies in fault data, thus revealing more in-depth information for transfer learning.
(4) An experiment is designed to validate the proposed method. Data of planetary gear and bearing were collected on the test rig across different operational conditions for validation. The result demonstrates that the method is effective and superior to other methods for the fault diagnosis of planetary gearboxes.

## 2. Theoretical Background

### 2.1. Representation of Nonstationary Operating Condition

In practical industrial applications, the operation of planetary gear systems is subject to variations in load and speed, leading to changes in the frequency components and amplitudes of vibration signals. This dynamic, nonstationary behavior makes it challenging to extract confident features from monitoring signals for fault diagnosis. To address this issue, time–frequency analysis methods are used to extract informative features from joint domain of time and frequency. Among these methods, the short-time Fourier transform (STFT) stands out for its computational efficiency and its ability to prevent cross-interference among signal components [24,25]. STFT divides time-sequential signals into segments using a window function of fixed length. Each segment is then transformed using the fast Fourier transform (FFT) to obtain a localized Fourier spectrum, retaining both time and frequency information. This method is particularly effective for analyzing nonstationary signals. It provides a clear representation of how frequency constitution evolves over time. The mathematical expression is given by

(1) $$x_{\mathrm{STFT}}(t,\omega )=\int_{-\infty }^{\infty }f(t)g(t-\tau )\exp(-j\omega t)dt,$$

where *f*(*t*) represents the time-series signal; *g*(*t* − *τ*) is a window function concentrated at a time instance *τ*. The STFT is a Fourier transform that is associated with the signal *f*(*t*) and the window function *g*(*t* − *τ*).

### 2.2. Feature Learning Based on Residual Algorithms

**Figure 2:**
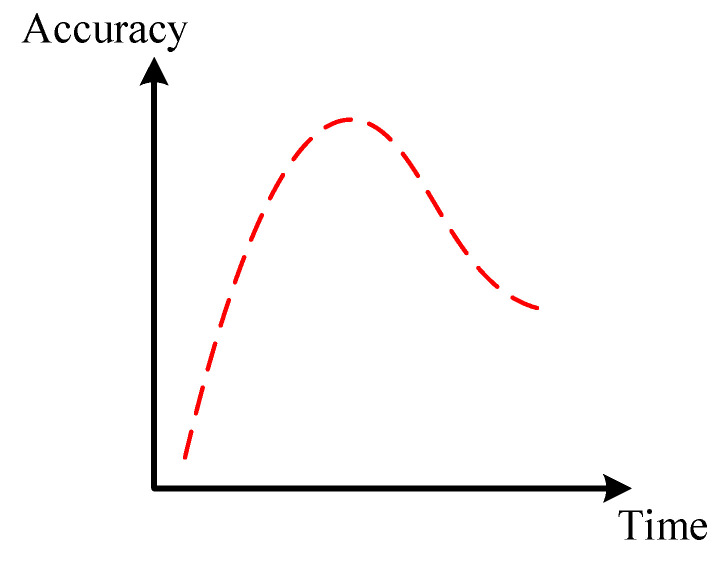
Overfitting during deep network training.

Features extracted through time–frequency methods are fed into deep convolutional networks to learn intrinsic time–frequency patterns. As the time–frequency representation is high-dimensional and the available data are often limited, they pose challenges to modeling for the fault diagnosis of planetary gearboxes [26]. While increasing the number of convolutional layers can theoretically enhance the ability of feature extraction, it also increases the risk of overfitting. This can result in an initial improvement in diagnostic performance, followed by a sharp decline, as illustrated in Figure 2. Additionally, deeper networks may face training issues, such as gradient vanishing or explosion. They affect stability and model performance.

**Figure 3:**
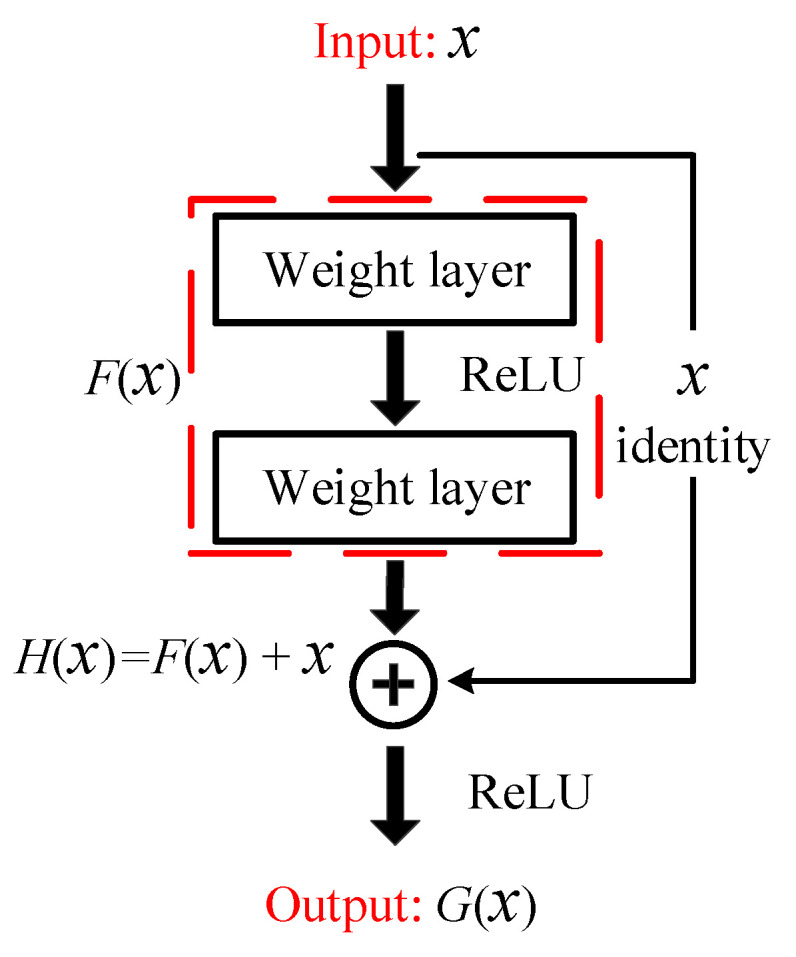
Schematic structure of residual block.

ResNets are particularly suitable to extract deep features from small sample data of planetary gearbox due to its unique network architecture and its ability to address the vanishing gradient problem through residual connections [27]. Figure 3 illustrates the core structure of ResNets, namely the residual block. They allow deep networks to be effectively trained by learning identity mappings, ensuring that original input information is preserved and complex patterns can be captured. The inclusion of shortcut connections helps maintain the gradient flow, enabling deeper architectures without degradation. This improves the extraction of fine-grained fault features from non-stationary vibration signals of planetary gearbox under varying loads and speeds.

In ResNets, the final output, *G*(*x*) is obtained by applying the activation function to the sum of the input x and the residual mapping *F*(*x*). Convolutional kernels act as filters, converting time–frequency representations into feature matrices. ReLU activation is then applied for nonlinear transformation. To prevent redundant computations, pooling layers are used for down-sampling. This process refines the high-level features, enhancing the ability to diagnose faults in planetary gearbox systems. The mathematical operation within the residual block is expressed as

(2) $$x_{j}^{l}=\max(0,\;\sum_{i}w_{ij}^{l}*x_{i}^{l-1}+b_{j}^{l}),$$

(3) $$x_{j}^{l}(h_{\max})=\underset{(i-1)W+1\leq h\leq iW}{\max}x_{j}^{l}(h),$$

where **x** denotes the value of a node in the convolutional layers; **w** represents the weight matrix; **b** refers to the bias; max( ) signifies the ReLU activation function; *W* denotes the pooling operation; and *h* encompasses all the feature values within the local range.

### 2.3. Domain Adversarial Training

**Figure 4:**
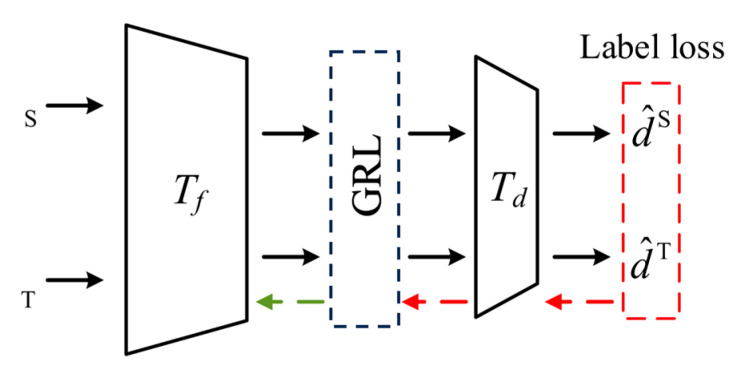
Domain adversarial training process.

Traditional adaptation methods typically rely on a fixed feature projection between the source and target domains, aiming to create a shared feature space for effective transfer learning. However, this approach often struggles to account for significant differences in feature distributions between two domains. To better handle this issues, domain adversarial training [22] is employed. This method encourages the model to generate features that are indistinguishable between the source and target domains, reducing discrepancies and improving cross-condition fault diagnosis. A domain discriminator $T_{d}$ distinguishes between source and target tasks, while a feature extractor $T_{f}$ works to confuse the discriminator by making it harder to differentiate between domain features. As shown in Figure 4, the parameters of the domain discriminator $\theta_{d}$ are optimized by minimizing discriminator loss, while a gradient reversal layer (GRL) inverts the loss gradient to optimize the feature extractor’s parameters $\theta_{f}$ by maximizing the discriminator loss. This adversarial training process continuously adjusts the model using the domain label loss *L_d_* and integrates both the discriminator and label losses in the overall objective function.

(4) $$L(\theta_{f},\theta_{d})=E_{x_{i}\in (D_{S}\cup D_{T})}[L_{d}(T_{d}(T_{f}(x_{i},\theta_{f}),\theta_{d}),d_{i})],$$

where *d_i_* denotes the domain label for sample *x_i_*, and $D_{S}$ and $D_{T}$ denote the source and target tasks, respectively.

### 2.4. Domain Confusion Training

**Figure 5:**
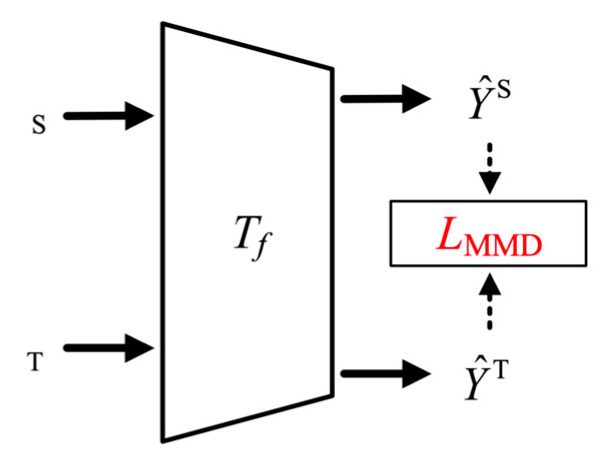
Domain confusion training process.

In the fault diagnosis of planetary gearboxes, correlations between vibration signals of different fault categories exist. Ignoring these correlations can lead to migration bias during model training. Thus, this paper adopts domain confusion training to adaptively assess and minimizes the feature distribution distance between source and target tasks. This ensures that the model remains responsive to variations in the target domain and improves its generalization across different operating conditions. A distribution distance metric, maximum mean discrepancy (MMD) [21], is used to calculate adaptation loss between the source and target domains ($D_{S}$ and $D_{T}$), as illustrated in Figure 5. MMD quantifies the difference of feature distributions, which is computed as

(5) $$\hat{d}(X_{S},\;X_{T})={\left\|\frac{1}{n_{S}}\sum_{i=1}^{n_{S}}\phi (x_{i})-\frac{1}{m_{T}}\sum_{j=1}^{m_{T}}\phi (x_{j})\right\|}_{H}^{2},$$

where H denotes the Hilbert space used to calculate the distribution distance, $\phi \left(\cdot \right)$ denotes the feature value of sample *x*, $n_{S}$ and $m_{T}$ denote the number of samples in the source and target tasks, respectively.

## 3. Subdomain Distribution Adversarial Adaptation Framework

### 3.1. Transfer Framework Design

**Figure 6:**
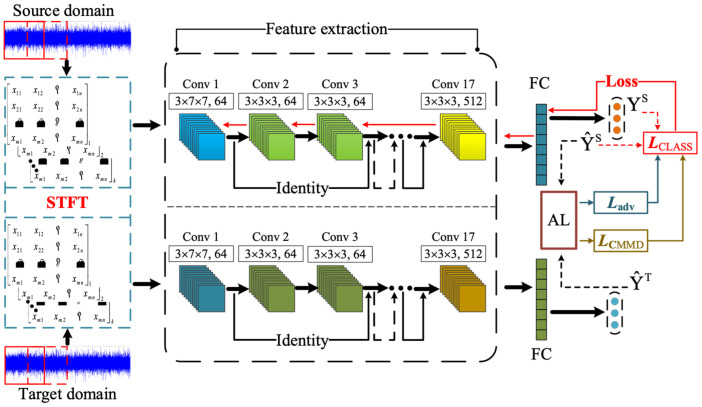
Transfer diagnostic framework for SDAA.

The fault data of planetary gearboxes often involves missing label information. Without sufficient labels, the model struggles to accurately map feature information to fault categories. To address this issue, pseudo-labeling techniques is adopted. The method iteratively refines its feature by gradually aligning the feature distributions more closely. This technique can leverage the target domain data more effectively, even in the absence of true labels. Additionally, the complexity of measuring feature differences increases in multi-category fault diagnosis tasks. When there are significant differences in data distributions among subclass faults between source and target tasks, assessing the marginal distribution differences becomes critical. Conversely, when marginal distributions are similar, it becomes important to evaluate and extract fault correlation transfer information. As illustrated in Figure 6, a novel diagnostic framework incorporating subdomain distribution adversarial adaptation is developed to identify the health states of planetary gearboxes. The framework adopts STFT to convert time-series vibration signals into time–frequency matrices. The ResNet18 is employed to extract deep features from time–frequency representations of signal. The framework then transforms the extracted time–frequency features from both source and target tasks into fault-related feature vectors. These vectors are used in an adaptation layer to adversarially evaluate subclass fault feature differences, rather than bridging these differences by retraining. Furthermore, conditional distribution adaptation is utilized to evaluate subclass fault-related information across tasks, thereby improving the precision of fault-related transfer information and avoiding feature distribution confusion between different faults.

### 3.2. Design of Loss Function

The proposed framework processes vibration signal data through several iterative operations involving convolutional and pooling layers. These layers operate together to extract features from signals with a collection of matrix transformation, which can be mainly denoted by Equations (2) and (3), Then the softmax function can be connected to the last layer of network for fault identification with a probability distribution. It can be defined as follows:

(6) $$\left[\begin{array}{c} p_{1}(x)\\ p_{2}(x)\\ \vdots \\ p_{c}(x) \end{array}\right]=\frac{\exp(x_{c})}{\sum_{i=1}^{C}\exp(x_{i})},$$

where *p*( ) denotes the probability distribution of category *c* in the multi-category diagnostic task, and *C* denotes the total number of fault categories. The cross-entropy loss function is utilized to optimize the network. By minimizing this loss between the predicted and actual labels of fault samples, the parameter of the network can be calculated for fault diagnosis. The function can be mathematically expressed as

(7) $$Loss\left(x,y\right)=-\frac{1}{N}\sum_{i=1}^{N}\sum_{c=1}^{C}y_{j}^{c}{\log\hat{y}}_{j}^{c},$$

where y and $\hat{y}$ indicates the true and predicted label of sample *x*, N denotes the number of samples.

The backpropagation algorithm is used to for model optimization. The model optimization in the source domain can be given by

(8) $$\arg\underset{w}{\min}\sum_{i}\sum_{l}L(y_{i}^{s},f(x_{i}^{s},w_{l}^{s})),$$

where *w* is the weight parameter of the *l*-th layer.

A term of loss function that measures the differences between sub-class faults within the source and target tasks are designed. In the adaptation layer, the mean loss of the local domain is calculated as the domain adversarial loss *L*_adv_ and expressed as

(9) $$L_{\mathrm{adv}}=\frac{1}{n_{s}+m_{t}}\sum_{c\in C}\sum_{x_{i}\in D_{S}\cup D_{T}}L_{d}^{c}(T_{d}^{c}(p_{i}^{c}(T_{f}(x_{i}))),d_{i}),$$

It quantifies the global correlation of transferred information from different tasks. In theory, the data distributions of identical faults across different tasks should be identical. To achieve this alignment, conditional distribution adaptation is utilized. Conditional maximum mean discrepancy (CMMD) [28] is employed to minimize this distribution distance across domains. It learns the shared structures or patterns that can be transferred from the source-domain data to the target-domain data of planet gearbox, thereby improving the feature alignment process. Features of different faults can be aligned more accurately in subclass level by considering the relationships between features within each fault in sub-class level. The conditional distribution adaptation loss *L*_CMMD_ involves using Equation (5) to determine the degree of alignment between distributions for each fault category.

(10) $$L_{\mathrm{CMMD}}(X_{S},X_{T})=\frac{1}{C}{\sum_{c}\left\|\frac{1}{n_{S}^{c}}\sum_{x_{i}^{c}\in D_{S}}^{n_{S}^{c}}\phi (x_{i})-\frac{1}{m_{T}^{c}}\sum_{x_{j}^{c}\in D_{T}}^{m_{T}^{c}}\phi (x_{j})\right\|}_{H}^{2},$$

The overall objective is designed in terms of *L*_adv_ and *L*_CMMD_ for parameter optimization, and it is denoted as

(11) $$\arg\underset{\theta_{f},\theta_{d}}{\min}(L_{c}^{D_{S}}(\hat{y}(x_{i}^{c},\theta_{f}),y_{i}^{c})+\lambda L_{\mathrm{joi}}^{D_{S}\cup D_{T}}(L_{\mathrm{CMMD}}(x_{i}^{c},\theta_{f}),L_{\mathrm{adv}}(x_{i}^{c},\theta_{f},\theta_{d}))),$$

The detailed training process of the SDAA diagnosis method is listed in Table 1.

## 4. Experimental Validation and Result Analysis

### 4.1. Experimental Setup

**Figure 7:**
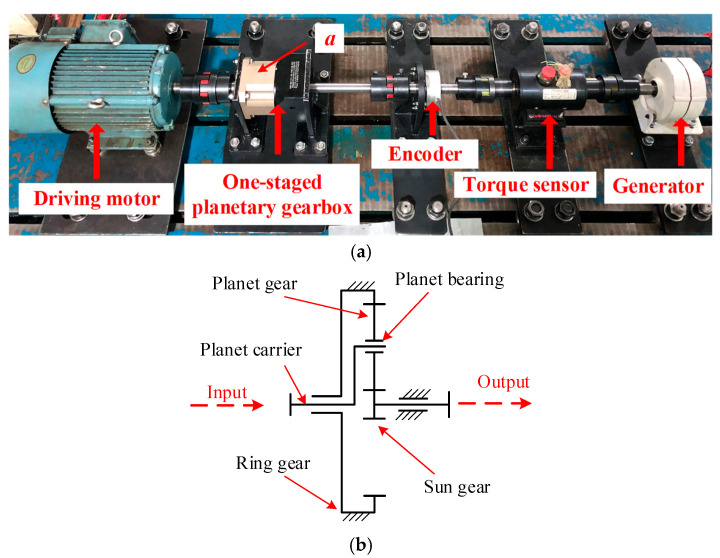
Test rig of one-staged planetary gearbox. (**a**) Experimental test rig; (**b**) diagram of the gearbox structure.

**Figure 8:**
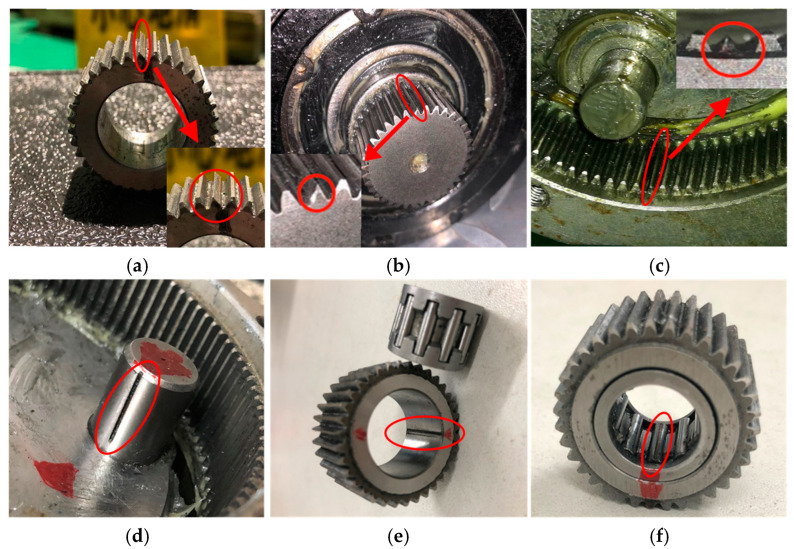
Damage parts in planetary gearboxes. (**a**) Planet gear fault; (**b**) sun gear fault; (**c**) ring gear fault; (**d**) inner race fault; (**e**) outer race fault; (**f**) rolling element fault.

A single-stage planetary gear system experimental platform, as shown in Figure 7a, was constructed to collect data for method validation. This test rig uses a PLX120 planetary gearbox, with a ratio of 4, to simulate wind turbine operations. The gearbox acts as a speed increaser. A three-phase induction motor provides power to drive the planetary carrier shaft. The output shaft of gearbox is linked to an AC permanent magnet generator, which functions as a load to dissipate electrical energy through a resistance box during operation. The specific parameters of the motor and the generator are given in Table 2. A rotary encoder and a torque sensor are positioned between the gearbox and the generator to measure rotational speed and torque, respectively. An accelerometer was positioned on the upper surface (acquisition point “a” in Figure 7a of the gearbox housing to accurately capture vibration signals for monitoring and fault diagnosis. The brand and model of accelerometer are PCB and 623C01, respectively. The frequency range is 0.8–15,000 Hz. A structural diagram of the PLX120 planetary gearbox is depicted in Figure 7b, with the detailed configuration of the gears and bearings listed in Table 3 and Table 4. Fault was simulated by chipping on the gear and bearing, and the details are depicted in Figure 8. The fault information is provided in Table 5.

### 4.2. Experimental Dataset

**Figure 9:**
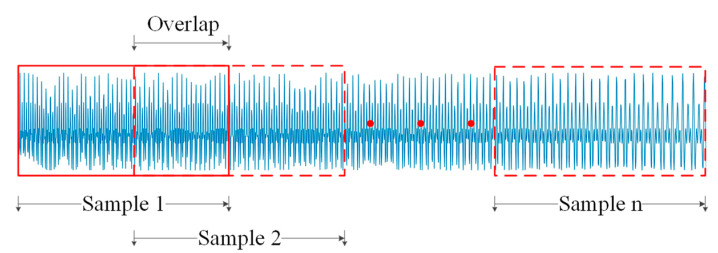
Sample division diagram in vibration signal.

Signals were sampled by the accelerometer at 20,480 Hz for 60 s, then resampled to 8192 Hz to reduce high-frequency interference and computational load. The resampled vibration signal was divided into overlapping slices, creating subsamples with 4096 data points each, covering a 0.5 s time span (Figure 9). 394 samples were generated for each data category as the source and target domain datasets. All 394 source domain samples and 78 target domain samples were used for training, while the remaining target domain samples were reserved for testing.

**Figure 10:**
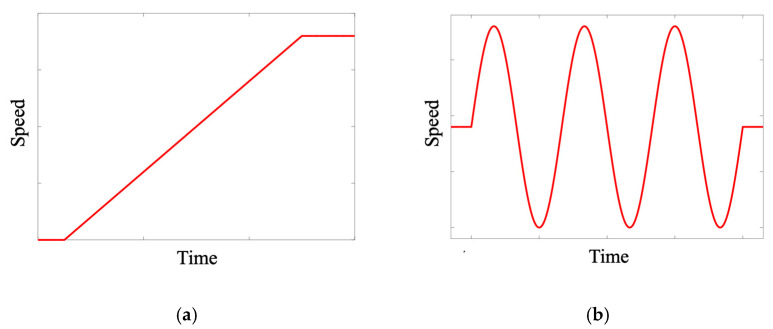
Motor speed curve under two time-varying mode. (**a**) Linearity; (**b**) sinusoidal.

The first dataset used for method validation comprises four distinct gear health states: normal condition (NC), planetary gear fault (PGF), sun gear fault (SGF), and ring gear fault (RGF). For each of these gear states, vibration signals were collected under two different motor speed conditions, with detailed configurations shown in Table 6. The first motor speed mode linearly accelerates from 0 rpm to 720 rpm (Figure 10a), while the second mode exhibits sinusoidal periodic variations (Figure 10b). This sinusoidal pattern effectively includes both nonlinear time-varying speed conditions, characterized by small time-scale acceleration and deceleration, and periodic time-varying speed conditions, characterized by large time-scale speed variations. When the motor speed is held constant, the generator torque remains steady. When the motor speed varies in a sinusoidal manner, the generator torque responds by adjusting inversely to the speed changes. This inverse adjustment ensures that the load torque is regulated according to the varying motor speed, maintaining the balance and performance of the system. Based on different motor speeds, nine transfer diagnostic tasks were designed for gear fault diagnosis, as detailed in Table 7.

The second dataset used for method validation comprises four distinct planetary bearing faults: normal (NC), inner race fault (IRF), outer race fault (ORF), and rolling element fault (REF). Likewise, experiments on the planet bearings for each fault type were conducted under two distinct time-varying motor speeds. The detailed speed configurations are given in Table 8. Based on the different motor speeds, a total of nine transfer diagnostic tasks were designed for this dataset. The specific details of these transfer diagnostic tasks are outlined in Table 8.

### 4.3. Method Validation and Diagnostic Results

To validate the reliability of the proposed method, the state-of-the-art methods were compared to SDAA for fault diagnosis under cross conditions, which includes ResNet18 [27], deep adaptation network (DAN) [21], deep domain adversarial network (DDAN) [22], and dynamic adversarial adaptation network (DAAN) [23]. All experimental analyses utilized ResNet18 as the base feature extractor within the PyTorch 1.3 framework. For each method, ten trials were conducted for each task to reduce randomness. The calculation was completed within 3.6 s for each trial. The performance of method was assessed by the metrics of mean and standard deviation.

(1) Fault Diagnosis of Planetary Gear

**Figure 11:**
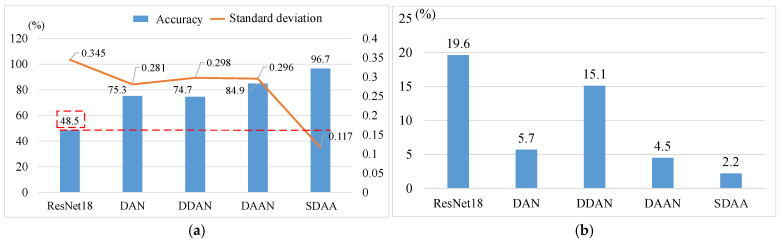
Comparison of diagnostic results for different methods. (**a**) Method Performance; (**b**) accuracy variation.

Table 9 presents the results of different methods for fault diagnosis of gear under cross conditions. SDAA demonstrates superior performance with an average diagnostic accuracy of 96.7% and a standard deviation of 0.117. By contrast, ResNet18 exhibits a lower average accuracy of 48.5% and a higher standard deviation of 0.345. The comparison underscores the effectiveness of SDAA in achieving reliable and consistent diagnostic results under cross condition in fault diagnosis. As illustrated in Figure 11a, adaptation methods improve diagnostic results, with SDAA obtaining the highest improvement (48.2%) and the lowest standard deviation, indicating consistent performance across multiple analyses. Additionally, Figure 11b demonstrates stability of different methods across various tasks. Compared to other methods, SDAA exhibits a variability of only 2.2% in diagnostic results between tasks. SDAA focuses not only on independently evaluating the marginal distribution differences between source and target tasks, but also the correlation among different subclass fault data. This results in a decrease in feature bias. The method can capture the nuanced relationships between subclass faults successfully. Consequently, SDAA enhances the overall performance of fault diagnosis tasks.

**Figure 12:**
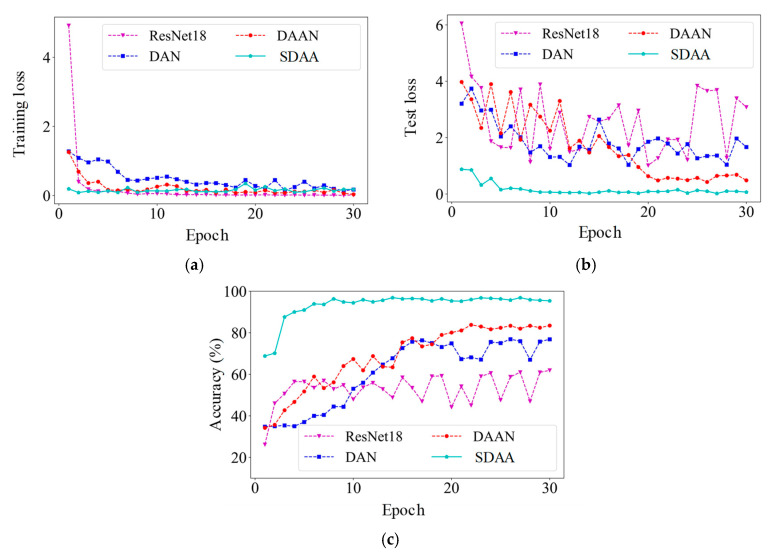
The convergence of training on the V_in_-V_3_ task. (**a**) Training loss; (**b**) test loss; (**c**) accuracy.

**Figure 13:**
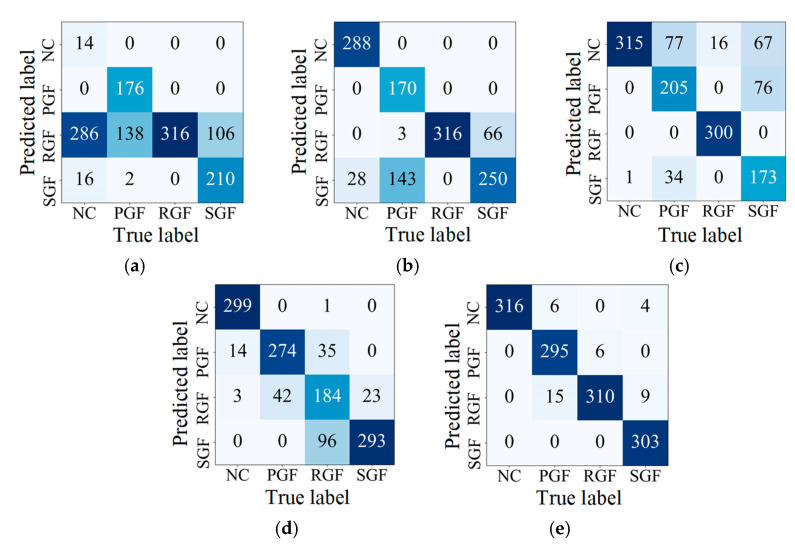
Fault diagnosis performance on the V_in_-V_3_ task. (**a**) ResNet18; (**b**) DAN; (**c**) DDAN; (**d**) DAAN; (**e**) SDAA.

Using the V_in_-V_3_ task as an example, Figure 12 illustrates the training process for various methods. In Figure 12a, all four methods show convergence during training. However, the losses for DAN, DAAN, and SDAA are higher than those for ResNet18, reflecting the greater impact of adaptation loss between the source and target tasks on model training. Figure 12b,c demonstrate that SDAA achieves faster test loss convergence, lower overall test loss, and higher diagnostic accuracy compared to the other methods. Additionally, SDAA maintains stability throughout training, with no significant fluctuations, indicating its effectiveness in evaluating data differences between the V_in_ and V_3_ conditions. This enables SDAA to extract more in-depth feature information from the source task. The confusion matrix in Figure 13 highlights the diagnostic performance of various methods in classifying data across different health states. From the matrices, it is evident that SDAA outperforms the other methods in classification accuracy. This comparison underscores the superiority of SDAA in the fault diagnosis of planetary gearboxes.

(2) Fault Diagnosis Results of Planetary Bearing

**Figure 14:**
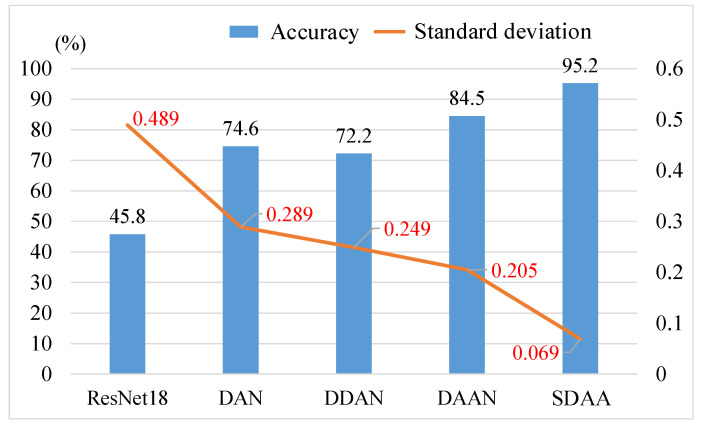
Comparison of diagnostic results for different methods.

**Figure 15:**
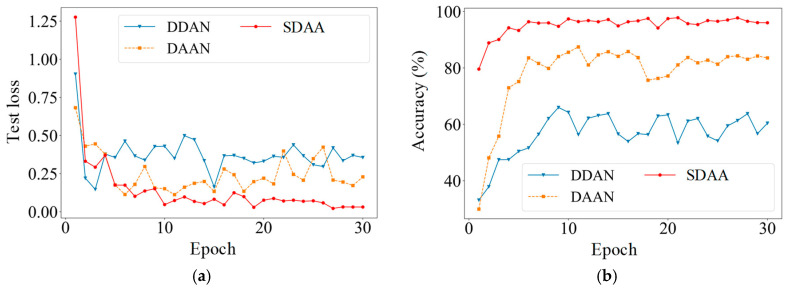
The convergence of adversarial adaptation methods on the B_in_-B_1_ task. (**a**) Test loss; (**b**) accuracy.

Table 10 presents the results of different methods for fault diagnosis of planetary bearings under cross conditions. As depicted in Figure 14, SDAA achieves the highest average result (95.2%) and the lowest standard deviation (0.069), indicating superior diagnostic performance in this task. This highlights the importance of effectively evaluating data discrepancies of sub-class data under cross condition. Figure 15 illustrates the test loss and diagnostic accuracy of three adversarial adaptation methods during the training of the diagnostic task B_in_-B_1_. In Figure 15a, the test losses for DDAN and DAAN fluctuate significantly and are consistently higher than those for SDAA. Likewise, Figure 15b reveals that the diagnostic accuracies of DDAN and DAAN are unstable during training. In contrast, SDAA exhibits the most stable performance throughout the entire training process.

In the fault diagnosis of planetary gearboxes, the proposed method excels other methods, as it integrates the adversarial training mechanism that specifically evaluates and aligns the feature distributions of the same fault types between the source and target domains. Moreover, the method incorporates conditional distribution adaptation, which enhances the model’s ability to correlate different sub-class faults within each domain. In planetary gearboxes, various sub-class faults exhibit subtle differences in their signal patterns. Conditional adaptation helps the model not only recognize these sub-classes but also establish relationships between them, even if they appear differently in the source and target domains. By addressing both the alignment of specific fault types and the correlations between sub-class faults, the proposed method improves the model’s ability to generalize across different conditions. This leads to more reliable fault diagnosis in planetary gearboxes, ensuring that the model can accurately identify faults even in varying operational environments, ultimately improving its performance in real-world applications.

**Figure 16:**
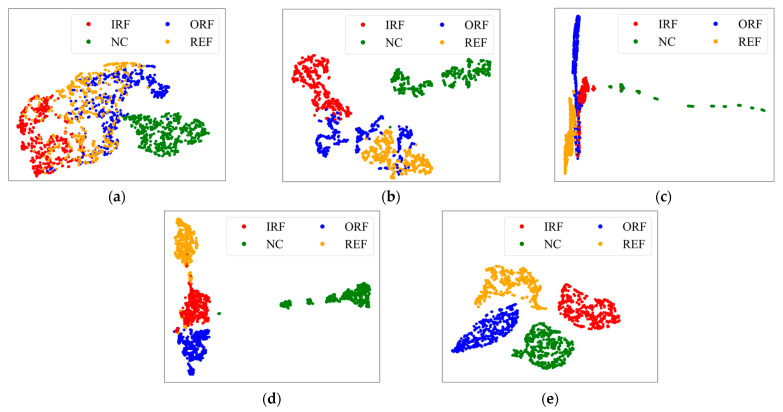
Feature visualization of different methods in task B_3_-B_1_. (**a**) ResNet18; (**b**) DAN; (**c**) DDAN; (**d**) DAAN; (**e**) SDAA.

Using the B_3_-B_1_ task as an example, Figure 16 visualizes the clustering of features using the t-SNE technique. In Figure 16a, samples from IRF, ORF, and REF fail to be accurately clustered by ResNet18, indicating its inadequacy for fault diagnosis of planetary bearings under cross-condition scenarios. In Figure 16b–d, features from different fault categories appear mixed, demonstrating that evaluating only the marginal distribution differences between source and target domain data are insufficient for accurate fault diagnosis. In contrast, Figure 16e shows that SDAA successfully clusters samples of the same fault together in the target domain. By bridging the gap between domains, SDAA offers a more reliable and robust approach to cross-condition fault diagnosis, outperforming other methods by focusing on both domain and sub-class data differences.

## 5. Conclusions

In practical engineering applications, correlations exist among different types of fault data in planetary gearboxes. Traditional methods often assume these fault data are independent, which is not the case. Additionally, planetary gearbox vibration signals contain complex frequency components that change over time. Relying solely on time-domain data fails to capture the inherent features of these signals. Both of these factors can significantly undermine the performance of fault diagnosis models, leading to less accurate or reliable results.

To address these issues, this paper proposes the SDAA method for planetary gearbox health state identification. The approach incorporates the STFT to compute the time–frequency representation of raw signals, which effectively captures intrinsic features without frequency overlap. Following this, a dual-stream network structure is employed to extract features from the time–frequency representations of both source and target tasks.

The method adopts adversarial training mechanism to evaluate the feature distribution differences between corresponding faults in the source and target domains. At the same time, conditional distribution adaptation strengthens the correlations between different subclass faults, improving the model’s ability to generalize to the target task’s data distribution.

Finally, the proposed method is validated through multiple cross-condition fault diagnosis scenarios on experimental data of planetary gearboxes. Comparative studies show that SDAA significantly enhances diagnostic performance, even with limited data samples for model training. This proves SDAA a practical and effective solution for planetary gearbox fault identification under varying operational conditions.

## Figures and Tables

**Table 1:** Training steps for SDAA.

**Input**: Given source domain data pairs $D_{S}={\left\{x_{i}^{S},y_{i}^{S}\right\}}_{i=1}^{n}$ and target domain data $D_{T}={\left\{x_{j}^{T}\right\}}_{j=1}^{m}$.
**Output**: The well-trained model.
**Begin**
1: Configure the adaptation layer in the diagnostic framework.
2: Randomly initialize the ResNet18 parameters.
**Training**
**for** epoch **in** epochs **do**
3: Simultaneously extract the feature information from the source domain data $D_{S}$ and target domain data $D_{T}$.
4: Computing the classification loss in the source task $D_{S}$, $L_{c}^{D_{S}}$.
5: Calculate the adversarial loss $L_{\mathrm{adv}}$ between $D_{S}$ and $D_{T}$.
6: Calculate the conditional adaptation loss $L_{\mathrm{CMMD}}$ between $D_{S}$ and $D_{T}$.
7: Fine-tuning the classification loss $L_{c}^{D_{S}}$ by adversarial loss $L_{\mathrm{adv}}$ and adaptation loss $L_{\mathrm{CMMD}}$.
8: Updating network weight parameters by back propagation with updated loss
**end for**
**Until** The loss in the target task converges or the training epochs reach.

**Table 2:** Parameters of induction motor and permanent magnet generator.

	**Induction Motor**	**Generator**
Rated power	4 kW	200 W
Rated voltage	380 V	24 V
No. of slots	36	39
No. of pole pairs	2	6

**Table 3:** Gear configuration parameters.

**Gear Type**	**Planet**	**Sun**	**Ring**
No. of gear teeth	35 (3)	36	108

Note: no. of planet gear in brackets.

**Table 4:** Planetary bearing configuration parameters.

**Diameter of Pitch Circle (mm)**	**Diameter of Rollers (mm)**	**No. of Rollers**	**Contact (°)**
19.5	3.5	10	0

**Table 5:** Fault sizes of gears and planetary bearings.

	**Length (mm)**	**Width (mm)**	**Depth (mm)**
Sun gear	16.3	0.2	0.8
Planet gear	17	0.2	0.8
Ring gear	17.5	0.2	0.8
Inner race (planet pin)	18	1	0.5
Outer race (planet bore)	17	1	0.5
Rolling element	11.5	1	0.5

**Table 6:** Motor speed configuration in gear fault experiments.

**Time-Varying Mode**	**Range of Motor Speed** **(rpm** **)**	**Symbol**
Linearity	0~720	V_in_/B_in_
Sinusoidal	150~330	V_1_/B_1_
	390~570	V_2_/B_2_
	510~690	V_3_/B_3_

**Table 7:** Transfer diagnosis task of gear faults.

	**Motor Speed (rpm)**		**Transfer Tasks**	**Health Status**
	**Source Domain**	**Target Domain**		
1	0~720	150~330	V_in_–V_1_	NC PGF RGF SGF
2		390~570	V_in_–V_2_	
3		510~690	V_in_–V_3_	
4	150~330	390~570	V_1_–V_2_	
5	390~570	150~330	V_2_–V_1_	
6	150~330	510~690	V_1_–V_3_	
7	510~690	150~330	V_3_–V_1_	
8	390~570	510~690	V_2_–V_3_	
9	510~690	390~570	V_3_–V_2_	

**Table 8:** Transfer diagnosis task of planet bearing faults.

	**Motor Speed (rpm)**		**Transfer Tasks**	**Health Status**
	**Source Domain**	**Target Domain**		
1	0~720	150~330	B_in_-B_1_	NC IRF ORF REF
2		390~570	B_in_-B_2_	
3		510~690	B_in_-B_3_	
4	150~330	390~570	B_1_-B_2_	
5	390~570	150~330	B_2_-B_1_	
6	150~330	510~690	B_1_-B_3_	
7	510~690	150~330	B_3_-B_1_	
8	390~570	510~690	B_2_-B_3_	
9	510~690	390~570	B_3_-B_2_	

**Table 9:** Diagnostic results of different methods in gear fault diagnosis.

	**ResNet18**	**DAN**	**DDAN**	**DAAN**	**SDAA**
V_in_-V_1_	59.5 ± 0.392	76.2 ± 0.232	70.4 ± 0.349	83.7 ± 0.295	95.3 ± 0.126
V_in_-V_2_	57.5 ± 0.329	77.3 ± 0.218	68.5 ± 0.327	86.7 ± 0.236	96.5 ± 0.096
V_in_-V_3_	56.4 ± 0.352	75.9 ± 0.187	77.2 ± 0.283	82.6 ± 0.228	95.9 ± 0.118
V_1_-V_2_	43.3 ± 0.285	76.2 ± 0.262	74.7 ± 0.274	83.4 ± 0.324	97.2 ± 0.112
V_2_-V_1_	44.9 ± 0.314	75.8 ± 0.278	73.5 ± 0.261	83.6 ± 0.332	96.8 ± 0.106
V_1_-V_3_	39.8 ± 0.402	72.7 ± 0.365	71.3 ± 0.282	85.8 ± 0.343	97.5 ± 0.130
V_3_-V_1_	40.3 ± 0.336	71.6 ± 0.322	70.5 ± 0.243	84.9 ± 0.349	97.1 ± 0.112
V_2_-V_3_	46.7 ± 0.347	75.4 ± 0.349	83.6 ± 0.358	86.3 ± 0.273	96.9 ± 0.121
V_3_-V_2_	48.3 ± 0.351	76.5 ± 0.318	82.3 ± 0.302	87.1 ± 0.285	97.3 ± 0.132
**Average**	**48.5** ± **0.345**	**75.3** ± **0.281**	**74.7** ± **0.298**	**84.9** ± **0.296**	**96.7** ± **0.117**

**Table 10:** Method comparison for fault diagnosis of planet bearing.

	**ResNet18**	**DAN**	**DDAN**	**DAAN**	**SDAA**
B_in_-B_1_	53.3 ± 0.435	74.2 ± 0.341	65.4 ± 0.252	87.6 ± 0.284	93.6 ± 0.106
B_in_-B_2_	51.9 ± 0.542	75.3 ± 0.357	63.5 ± 0.231	83.5 ± 0.231	95.8 ± 0.083
B_in_-B_3_	49.8 ± 0.536	75.9 ± 0.226	68.2 ± 0.257	82.8 ± 0.198	94.7 ± 0.035
B_1_-B_2_	42.4 ± 0.528	75.1 ± 0.216	73.7 ± 0.162	82.1 ± 0.302	95.9 ± 0.052
B_2_-B_1_	43.5 ± 0.484	74.2 ± 0.252	74.3 ± 0.151	81.7 ± 0.294	94.6 ± 0.084
B_1_-B_3_	39.6 ± 0.455	70.8 ± 0.256	67.4 ± 0.201	85.5 ± 0.132	94.8 ± 0.108
B_3_-B_1_	40.2 ± 0.521	69.9 ± 0.218	68.8 ± 0.442	84.3 ± 0.118	95.7 ± 0.043
B_2_-B_3_	45.3 ± 0.442	77.3 ± 0.351	84.2 ± 0.294	86.3 ± 0.154	96.7 ± 0.057
B_3_-B_2_	46.5 ± 0.458	78.8 ± 0.386	84.7 ± 0.259	86.4 ± 0.134	95.2 ± 0.052
**Average**	**45.8** ± **0.489**	**74.6** ± **0.289**	**72.2** ± **0.249**	**84.5** ± **0.205**	**95.2** ± **0.069**

## Data Availability

The data presented in this study are available on request from the corresponding author.
